# Validation of a German version of the Boredom Proneness Scale and the Multidimensional State Boredom Scale

**DOI:** 10.1038/s41598-024-53236-4

**Published:** 2024-02-05

**Authors:** Katharina Zerr, Johannes P.-H. Seiler, Simon Rumpel, Oliver Tüscher

**Affiliations:** 1 Department of Psychiatry and Psychotherapy, University Medical Center of the Johannes Gutenberg University Mainz, Untere Zahlbacher Straße 8, 55131 Mainz, Germany; 2 Institute of Physiology, Focus Program Translational Neurosciences, University Medical Center of the Johannes Gutenberg University Mainz, Hanns-Dieter-Hüsch-Weg 19, 55131 Mainz, Germany; 3 Leibniz Institute for Resilience Research, Wallstraße 7, 55122 Mainz, Germany; 4 Institute of Molecular Biology, Ackermannweg 4, 55128 Mainz, Germany

**Keywords:** Cancer, Immunology, Oncology

## Abstract

The scientific interest in boredom is growing over the past decades. Boredom has not only been linked to symptoms of psychopathology, but also shows a remarkable effect on individual behavior under healthy conditions. Current characterizations of boredom in humans mostly rely on self-report assessments which proved to faithfully reflect boredom in a vast range of experimental environments. Two of the most commonly used and prominent self-report scales in order to assess boredom are the Multidimensional State Boredom Scale (MSBS) and the Boredom Proneness Scale (BPS). Here, we present the German translations of both questionnaires and their validation. We obtained and analyzed psychometric data from more than 800 healthy individuals. We find that the German MSBS and BPS show vast congruence with their originals in respect to item statistics, internal reliability and validity. In particular, we find remarkable associations of state boredom and trait boredom with indicators of mental burden. Testing the factor structure of both questionnaires, we find supporting evidence for a 5-factor model of the MSBS, whereas the BPS in line with its original shows an irregular, inconsistent factor structure. Thus, we validate the German versions of MSBS and BPS and set a starting point for further studies of boredom in German-speaking collectives.

## Introduction

Boredom is an aversive mental state, arising from monotonous situations^1–5^ and cognitive disengagement^6,7^, with profound effects on human perception and behavior across cultures^8^. No experience demonstrates this as clearly as the pandemic restrictions of the past years all over the world. In this respect, chronical boredom has been identified as an unpleasant experience^1,9^, associated with negative mood and depressive symptoms in adults^10–12^ and in children^13^. Besides the experiential aspects, boredom was identified as a behavioral driving factor that prompts negative consequences, such as over-eating^14,15^ and alcohol consumption^16,17^. Importantly, if adequately managed, boredom can also has positive implications on individual behavior, by promoting the search for novel information and hence driving the exploration of one’s environment^2,3,18,19^.

Together, this highlights boredom as a fundamental, psychosocially relevant experience. However, the scientific studies on boredom represent a comparably young field^20,21^, demanding further characterizations of boredom in a variety of experimental contexts. For this purpose, various self-report assessments have been developed in order to measure *state boredom*—defined as a temporally limited experiece of boredom—as well as *trait boredom*—defined as the rather stable individual proneness to be bored^22^ (in this study, we use the terms *boredom proneness* and *trait boredom* synonymously). State boredom, on the one side, is typically regarded as a functional cognitive signal, indicating that a current environment does not offer sufficient information^3,23^ and cognitive stimulation^6^, hence promoting exploration^2^. Trait boredom on the other side, has been defined by the frequency of experiencing boredom and is thought to be mainly determined by an individual’s degree of agency^24^ and its attentional capabilities^1^. Despite a marked overlap between the concepts of state and trait boredom, their different characteristics require distinct psychometric assessments. Hence, multiple self-report scales have been developed in order to characterize the neuropsychological signatures of state and trait boredom specifically, each of them having a unique profile in respect to the operationalization and measured aspects of boredom (for a review see ref^22,25^). Nevertheless, two psychometric boredom scales have found predominant application in the past to investigate boredom as a state and trait: (i) The Multidimensional State Boredom Scale (MSBS)^26^ is a vastly applied self-report assessment of state boredom that shows high validity across different studies and samples^27–30^; (ii) The Boredom Proneness Scale (BPS)^31^ was developed early and applied broadly to characterize trait boredom under healthy and pathological conditions^32–34^. Both questionnaires, the MSBS and BPS, were established in English language and have mostly been used to study boredom in English-speaking collectives.

With the current study, we aim to widen the scope of psychometric boredom research by translating the MSBS and BPS into German language and validating these translations. Specifically, we collect cross-sectional MSBS ratings and BPS ratings from N = 883 healthy adult participants and analyze them in respect to the single item statistics of the questionnaires, internal consistency and convergent validity. We furthermore compare the dimensionality of the translated boredom questionnaires with their English originals by applying exploratory and confirmatory factor analyses.

We find similar characteristics between the German versions of the MSBS, BPS and their originals, indicating that the German MSBS and BPS can complement the toolkit of boredom measurements, thus facilitating studies on boredom in German-speaking populations.

## Results

### Questionnaires and demographic characterization of the experimental sample

In a first step, we translated the English original of the Multidimensional State Boredom Scale (MSBS)^26^ and Boredom Proneness Scale (BPS)^31^ into German (Supplementary Information). The translations were conducted by an independent, professional agency and were checked for congruency with the originals (Methods). Then, we recruited a sample of N = 883 healthy, adult participants that filled out the German versions of MSBS and BPS in uncontrolled home environments (see Supplementary Table 1 for demographic information). In addition, participants completed a battery of other psychometric self-report assessments in order to measure boredom-related constructs, such as impulsivity, willingness to take risks and depressive symptoms (for details see Methods, for measures of consistency see Supplementary Table 2). Participants were predominantly female (66.4%) and had an average age of 41.5 years (± SD 11.7).

### Validation of the German Multidimensional State Boredom Scale

In order to validate the translated version of the Multidimensional State Boredom Scale (MSBS), we first investigated the single item ratings of subjects (Supplementary Table 3). We found that all items were rated with medium inter-individual variance (standard deviation ranging from min. 1.142 to max. 1.786 on a 7-point Likert scale) and medium difficulty (a measure for noise in single-item ratings, ranging from min. 0.099 to max. 0.370), hence indicating reliable but differential responses across subjects^35^.

In a next step, we aimed to investigate the factor structure of the German MSBS, in order to unravel different dimensions that contribute to self-reported state boredom. Therefore, we conducted an exploratory factor analysis (EFA) for a random subsample of N = 291 participants (Methods). An unsupervised Velicer’s minimum average partial test (MAP test)^36^ to determine the dimensionality of the questionnaire suggested a 3-factor model for the German MSBS. For this 3-factor structure, most items showed a loading pattern that was clearly attributable to one of the factors (N = 291, R^2^: total: 0.49, Factor 1: 0.23, Factor 2: 0.13, Factor 3: 0.13; Supplementary Table 4). However, as the suggested 3-factor model deviates from the English original MSBS that was established with a 5-factor structure^26^, we conducted a comparative factor analysis assuming a 5-subscale structure. Here, we found higher levels of explained variance for the original 5-factor model (N = 291, R^2^: total: 0.56, Factor 1: 0.16, Factor 2: 0.12, Factor 3: 0.12, Factor 4: 0.10, Factor 5: 0.06). To corroborate this observation, we performed a confirmatory factor analysis to compare both factor structures, using the data from N = 576 independent participants. In this comparative confirmatory factor analysis, we found that the original 5-factor model explained more of the variance in the observed data (Table 1), showing a higher comparative fit index as well as lower prediction errors^37^, hence supporting the original factor structure of the MSBS.

**Table 1:** Comparison of confirmatory factor analyses for a 3-factor vs. 5-factor model of the German MSBS.

Confirmatory factor analysis MSBS (N = 576)	CFI	AIC	RMSEA	SRMR	GFI
MSBS 3-factor (from MAP test and EFA)	0.875	51,560	0.083	0.060	0.791
MSBS 5-factor (original)	0.917	49,309	0.069	0.055	0.838

Based on a Velicer’s minimum average partial test suggesting a 3-factor structure for the German MSBS (conducted with a random subsample of N = 291 participants), we applied a confirmatory factor analysis and compared the goodness of the 3-factor structure against the original 5-factor structure (N = 576 remaining participants).

We find superiority of the original 5-factor structure for various parameters of model goodness.

CFI: Comparative Fit Index; AIC: Akaike Information Criterion; RMSEA: Root Mean Square Error of Approximation; SRMR: Standardized Root Mean Square Residual; DFI: Goodness of Fit Index; MAP test: Velicer’s minimum average partial test.

Based on this 5-factor model of the MSBS, we then compared the loadings of single questions on the model factors (Table 2). We found that the overall loading profile was similar to previous characterizations of the MSBS^26,28^, with however some incongruence in the Disengagement subscale (4 of 10 items of this subscale showed also high loadings on the Low Arousal factor). In order to test if an exclusion of these items with high cross-loadings significantly improves the model fit, we tested the goodness of a 5-factor model on a subset of our data with all ambiguous items removed that show high loadings on more than factor (Methods). The model fit of this shortened version of the German MSBS was comparable to the long version (N = 291, R^2^: total: 0.58, Factor 1: 0.16, Factor 2: 0.13, Factor 3: 0.11, Factor 4: 0.11, Factor 5: 0.06). Together, our findings indicate a wide conformity of the factor structure between the German MSBS and the English original.

**Table 2:** Confirmatory factor analysis of the German MSBS for a 5-factor model with loadings of single items.

MSBS item	Factor 1 (Diseng.)	Factor 2 (High Ar.)	Factor 3 (Inattent.)	Factor 4 (Low Ar.)	Factor 5 (Time P.)
2	0.208	0.192	0.151	**0.434**	0.269
7	0.288	0.172	0.106	**0.498**	0.316
9	0.227	0.310	0.174	**0.468**	0.140
10	**0.410**	0.079	0.191	**0.426**	0.377
13	0.282	**0.511**	0.312	0.303	0.132
17	**0.537**	0.219	0.269	0.336	0.200
19	**0.630**	0.193	0.148	0.259	0.233
22	**0.431**	0.366	0.316	0.194	0.188
24	**0.628**	0.306	0.183	0.313	0.153
28	**0.510**	0.288	0.206	0.398	0.337
5	0.109	**0.512**	0.226	**0.497**	0.148
12	0.094	**0.583**	0.257	0.271	0.234
14	0.197	**0.733**	0.301	0.188	0.067
21	0.284	**0.664**	0.284	0.062	0.136
27	0.158	**0.512**	0.106	0.285	0.219
3	0.166	0.236	**0.721**	0.161	0.106
16	0.181	0.364	**0.764**	0.226	0.112
20	0.244	0.397	**0.613**	0.205	0.180
23	0.379	**0.489**	**0.447**	0.129	0.125
4	0.183	0.289	0.155	**0.562**	0.144
8	0.114	**0.652**	0.126	**0.521**	0.178
15	0.193	**0.526**	0.158	**0.561**	0.155
25	0.242	0.342	0.174	**0.410**	0.228
29	0.196	0.106	0.095	**0.552**	0.168
1	0.114	0.069	0.082	0.047	**0.731**
6	0.096	0.117	0.076	0.299	**0.572**
11	0.177	0.146	0.110	0.284	**0.800**
18	0.139	0.164	0.088	0.198	**0.891**
26	0.170	0.202	0.096	0.119	**0.853**

Data from N = 576 participants.

The items are sorted according to the 5-subscale structure (Disengagement, High Arousal, Inattention, Low Arousal, Time Perception) suggested for the English original^26^.

High loadings > 0.4 are presented in bold letters.

In order to test the cross-item reliability of the German MSBS, we computed internal consistency, as measured by Cronbach’s α, for the full MSBS and its subscales^38^ (Table 3). For the subscales we found values of Cronbach’s α ranging from 0.841 (Low Arousal) to 0.904 (Time Perception), as well as a value of 0.95 for the MSBS sum score. These scores indicate good and reliable internal consistency of the German MSBS. In accordance with this, the different subscales of the MSBS showed robust inter-correlations as well as a marked association with the MSBS sum score (Supplementary Table 5).

**Table 3:** Internal consistency of the German MSBS.

	Number of items	Cronbach's α
MSBS sum score	29	0.950
MSBS-subscale disengagement	10	0.898
MSBS-subscale high arousal	5	0.858
MSBS-subscale low arousal	5	0.841
MSBS-subscale inattention	4	0.875
MSBS-subscale time perception	5	0.904

The internal consistency, as measured by Cronbach’s α, is computed for the data of N = 867 participants.

Lastly, we tested the convergent validity of the translated MSBS by correlating it with other boredom-related psychometric constructs (Table 4). We found a strong correlation of state boredom measured by the MSBS and its subscales with trait boredom measured by the BPS (N = 843, Spearman’s R = 0.724). Moreover, the MSBS sum score as well as its subscales were strongly positively correlated to measures of mental burden and symptoms of depression, anxiety and attention deficit hyperactivity disorder (MSBS sum score: Spearman’s R ranging from 0.571 to 0.669). Here, the highest correlation to psychopathologies was found for the High Arousal and Inattention subscales, whereas the Time Perception subscale only showed a weak association with mental health issues. Also, various dimensions of impulsivity, except risk affinity, were associated with increased MSBS ratings. All reported correlations were statistically robust against Bonferroni correction for multiple testing (*p* < 0.001). Thus, the correlation profile of the German MSBS to external psychometric constructs is equivalent to the original questionnaire and translations in other languages^26,28,29^.

**Table 4:** Correlation of the German MSBS and its subscales to other boredom-related psychometric constructs.

MSBS		BPS	GHQ-28	BDI-II	CAARS:S-L	STAI-Y2	I-8 Urg	I-8 Purp	I-8 End	I-8 Risk
N		843	859	862	814	855	862	862	862	862
Sum score	SpearmanR	**0.724**	**0.571**	**0.669**	**0.589**	**0.658**	**0.357**	**– 0.152**	**– 0.338**	– 0.058
	Sig. (*p*)	< 0.001	< 0.001	< 0.001	< 0.001	< 0.001	< 0.001	< 0.001	< 0.001	0.090
Diseng	SpearmanR	**0.595**	**0.470**	**0.591**	**0.530**	**0.589**	**0.344**	**0.145**	**0.333**	– 0.035
	Sig. (*p*)	< 0.001	< 0.001	< 0.001	< 0.001	< 0.001	< 0.001	< 0.001	< 0.001	0.303
High arousal	SpearmanR	**0.463**	**0.620**	**0.642**	**0.553**	**0.645**	**0.322**	**0.129**	**0.280**	**– 0.073**
	Sig. (*p*)	< 0.001	< 0.001	< 0.001	< 0.001	< 0.001	< 0.001	< 0.001	< 0.001	0.030
Low arousal	SpearmanR	**0.441**	**0.477**	**0.565**	**0.568**	**0.565**	**0.310**	**0.154**	**0.364**	**– 0.104**
	Sig. (*p*)	< 0.001	< 0.001	< 0.001	< 0.001	< 0.001	< 0.001	< 0.001	< 0.001	0.002
Inattent	SpearmanR	**0.463**	**0.566**	**0.656**	**0.511**	**0.651**	**0.328**	**0.134**	**0.310**	**– 0.074**
	Sig. (*p*)	< 0.001	< 0.001	< 0.001	< 0.001	< 0.001	< 0.001	< 0.001	< 0.001	0.029
Time percept	SpearmanR	**0.396**	**0.292**	**0.338**	**0.286**	**0.316**	**0.170**	**0.083**	**0.189**	– 0.001
	Sig. (*p*)	< 0.001	< 0.001	< 0.001	< 0.001	< 0.001	< 0.001	< 0.001	< 0.001	0.971

MSBS: Multidimensional State Boredom Scale; BPS: Boredom Proneness Scale; GHQ: General Health Questionnaire; BDI: Beck’s Depression Inventory; CAARS: Conner’s Adult ADHD Rating Scale; I: Impulsivity questionnaire with subscales for Urgency, Purpose, Endurance and Risk Affinity.

Significant correlations after Bonferroni correction for multiple testing are highlighted in bold letters.

### Validation of the German boredom proneness scale

In order to validate the German translation of the Boredom Proneness Scale^31^ (BPS), we pursued a similar approach as for the MSBS. First, we tested the single item statistics of the BPS and found that all items were rated with medium inter-individual variance (standard deviation ranging from min. 1.227 to max. 1.926) and medium difficulty (ranging from min. 0.131 to max. 0.610), similar to the MSBS translation (Supplementary Table 6).

Since the factor structure of the BPS has been shown to vary strongly across different studies^39^, we next sought to characterize the dimensionality of the German translation of the BPS with an exploratory factor analysis for a random subset of participants (N = 284 randomly chosen participants, Methods). We conducted a Velicer’s MAP test^36^ that suggested a 3-factor structure for the German BPS. Applying a factor analysis and comparing the loading patterns of single items on the three factors, we observed that multiple items were not well explained by the model structure, corresponding to the English original^40^ (N = 284, R^2^: total: 0.35, Factor 1: 0.13, Factor 2: 0.12, Factor 3: 0.09; Supplementary Table 7).

In order to quantitatively test the goodness of the 3-factor model, we then conducted a confirmatory factor analysis with the remaining dataset (N = 571 participants). We observed insufficiency in various measures of model goodness for the German BPS (comparative fit index CFI = 0.716; root mean square error of approximation RMSEA = 0.089; other fit indices are detailed in Table 5). This indicates that the German BPS has an irregular dimensionality that is hardly captured by simple factor models, in line with the diffuse factor structure of the English original^39–41^. For the purpose of tackling these structural limitations of the original BPS, prior studies proposed to cut the ambiguous items of the questionnaire^42^ and also remove the items that are reversed in wording^40^. Following the same approach, we removed all reverse worded items as well as those items with cross-loadings on multiple factors from the BPS dataset (Methods) and conducted an exploratory factors analysis to test if this adjustment could clarify the factor structure. Indeed, a Velicer’s MAP test suggested a single-factor structure for the adjusted BPS, which was supported by an exploratory factor analysis showing a similar fit as the more complex factor structure (N = 284, R^2^: total/Factor 1: 0.31). Together, this indicates that our German translation of the BPS is structurally similar to the English original, including its deficits in the factor structure, which however could be corrected by shortening the scale and removing ambiguous items.

**Table 5:** Confirmatory factor analysis for a 3-factor model of the German BPS.

Confirmatory factor analysis BPS (N = 571)	CFI	RMSEA	SRMR	GFI
BPS 3-factor (from MAP test and EFA)	0.716	0.089	0.085	0.804

Based on a Velicer’s minimum average partial test suggesting a 3-factor structure for the German BPS (random subsample of N = 284 participants), we applied a confirmatory factor analysis (data from N = 571 participants) revealing poor goodness of this model.

CFI: Comparative Fit Index; RMSEA: Root Mean Square Error of Approximation; SRMR: Standardized Root Mean Square Residual; DFI: Goodness of Fit Index; MAP test: Velicer’s minimum average partial test.

The internal consistency of the German BPS sum score, measured as Cronbach’s α^38^, was sufficient (Cronbach’s α = 0.863, N = 855 participants), indicating good reliability.

Furthermore, we compared the German BPS sum score to psychometric assessments of other boredom-related constructs and found highly significant, medium to high correlations (Table 6). In particular, we observed remarkable associations of BPS scores and measures of depressive symptoms, anxiety and impulsivity.

**Table 6:** Correlation of the German BPS to other boredom-related psychometric constructs.

		MSBS	GHQ-28	BDI-II	CAARS: S-L	STAI-Y2	I-8 Urg	I-8 Purp	I-8 End	I-8 Risk
BPS	SpearmanR	**0.724**	**0.445**	**0.560**	**0.539**	**0.632**	**0.379**	**–0.208**	**–0.397**	**–0.128**
	Sig. (*p*)	< 0.001	< 0.001	< 0.001	< 0.001	< 0.001	< 0.001	< 0.001	< 0.001	< 0.001
	N	843	847	850	802	843	850	850	850	850

BPS: Boredom Proneness Scale; MSBS: Multidimensional State Boredom Scale; GHQ: General Health Questionnaire; BDI: Beck’s Depression Inventory; CAARS: Conner’s Adult ADHD Rating Scale; I: Impulsivity questionnaire with subscales for Urgency, Purpose, Endurance and Risk Affinity.

Significant correlations after Bonferroni correction for multiple testing are highlighted in bold letters.

## Discussion

In this study, we validate the German translation of two widely used psychometric boredom assessments: the Multidimensional State Boredom Scale (MSBS) and the Boredom Proneness Scale (BPS). We find that the German translations of the questionnaires are structurally similar to the original versions and show similar correlation profiles to other boredom-related features. Hence, we set a cornerstone to standardize the assessment of state boredom and trait boredom in German-speaking societies.

For the study, we recruited participants from a study pool of individuals that was limited to an age between 18 and 60 years and furthermore comprised more female than male participants. These demographic characteristics, together with the uncontrolled conditions under which the self-report scales were rated, limit the generalizability of our findings, since groups of individuals with different demographic profiles or under different environmental conditions could deviate systematically in their self-reports of state and trait boredom. The original MSBS and BPS were established with data from students in young adult age^26,31^, hence showing a discrepancy to our sample. However, we observe that the general validation criteria of the German versions of the boredom questionnaires are similar to the English originals, indicating that boredom is prevalent across different ages^43,44^ and that the self-report scales reliably assess boredom across age categories.

Methodological drawbacks, such as e.g. dishonesty biases due to social desirability^45–47^, generally affect the validity of self-report scales. Here, the German boredom questionnaires used in this study have recently been associated with non-verbal, behavioral readouts of boredom^3^. In particular, state boredom measured by the German MSBS was found to positively correlate to monotony avoidance, a behavior that has been linked to boredom experience in various studies^4,48–50^. Thus, the MSBS and BPS show validity across different domains of probing boredom.

We observed some questionnaire-specific similarities and discrepancies between the original versions of the boredom questionnaires and their German translations. For the MSBS, our exploratory factor analysis suggested a 3-factor model of the questionnaire rather than the 5-factor model from the original^26^, suggesting latent factors in the originally proposed questionnaire structure. This discrepancy could be explained by alternative methods to estimate the number of underlying factors in an EFA: The original 5-factor EFA was based on the generation of questions for the MSBS in five different domains, whereas here we used a Velicer’s minimal average partial test to determine the number of factors. However, in a subsequent confirmatory factor analysis (CFA), we observed that the original 5-factor model was explaining our data better than the 3-factor model. Thus, the original 5-factor model of the MSBS seems appropriate also for the German translation and outperforms a simpler factor structure. The fact that some MSBS items in the CFA show a high loading on multiple subdimensions hints towards a partial overlap of subscales, especially of Disengagement and Low Arousal which share a substantial number of items. We also tested if an exclusion of the ambiguous items from the MSBS can enhance its fit to the factor model, which however did not lead to a relevant increase of explained variance.

Comparing the overall goodness of fit for the 5-factor model of the German MSBS with other studies that applied the MSBS, shows concordant measures of fit (CFI = 0.92 in this study, whereas CFI was distributed between 0.93 and 0.98 in other studies)^26,28–30^. Furthermore, these previous validation studies report an internal consistency of the MSBS (Cronbach’s α ranging from 0.89 to 0.98 for the MSBS sum score) that matches our findings (Cronbach’s α = 0.95). Lastly, also the correlation profile of German MSBS scores with depressive symptoms, anxiety, impulsivity and general mental burden is in line with prior correlation analyses^26–29^. Thus, the German MSBS is vastly congruent to applications of the English original.

Similarly, also the BPS sum score in our study was positively correlated with state boredom, different aspects of impulsivity, depression, anxiety as well as general mental burden, underlining the close relationship of trait boredom with mental health issues^51–53^.

However, the validity of the BPS in respect to its validity for measuring trait boredom has been questioned, due to inconsistent evidence regarding its factor structure^32,40,41^. For instance, the BPS has been proposed to comprise two^39,54–56^, four^57^, five^32^ or even eight factors^58^. Our results integrate into this picture, reporting an insufficient fit of the data with a 3-factor model that was suggested by a minimum average partial test in the context of an exploratory factor analysis. We observed a partial overlap of the 3-factor structure from our EFA with the commonly suggested 2-factor structure, separating trait boredom by aspects of internal and external stimulation problems^32,39,54,56^. Nevertheless, it has been shown that this dichotomic structure is confounded by the wording of single items in the scale and that the questionnaire structure can be homogenized to a one-factor model by removing those items^40^. Here, we replicate this finding, indicating that a shortened form of the German BPS could overcome some of the structural limitations of the full scale. In particular, excluding ambiguous and reverse worded items from the BPS promises suitable and valid tools for future studies on the specific characteristics of trait boredom and its neurocognitive underpinnings. Here, a clear conceptualization of trait boredom based on individual features, such as lacking agency^24^ and sense of meaning^9^, will be crucial to validly interpret the dimensionality of the shortened BPS. In line with these considerations, also shortened versions of the MSBS have been proposed^59,60^. Such condensed versions of boredom questionnaires could further widen the applicability of boredom assessments to different experimental conditions. We hope that our validation study of the full German BPS and MSBS translations paves the way for creating and validating similar short versions of the questionnaires to be applied in German-speaking collectives.

As all participants in our study independently filled out the questionnaires, the boredom ratings here were uncontrolled in respect to situational and environmental influences. In the future, it will be interesting to further apply the MSBS and BPS in defined experimental settings to test for systematic environmental factors that influence self-reports of boredom^61^. Due to the observed association with mental burdens and symptoms of psychopathology, especially a clinical context offers a field of promising and interesting application^53^.

Taken together, we present the translated versions of the BPS and MSBS to complement the tool set for studying state and trait boredom, in order to enable further investigations that deepen the understanding of boredom.

## Methods

The study was approved by the local ethics committee (Ethikkommission der Landesärztekammer Rheinland-Pfalz, processing numbers: 2018–13,164) and was conducted in accordance with the Declaration of Helsinki. Written informed consent was obtained from all participants of the study.

### Participants

In order to exploratively assess the link of boredom and psychopathological symptoms in a healthy sample, we randomly contacted approximately 1800 subjects from the pool of the *Gutenberg Brain Study* (https://lir-mainz.de/en/gutenberg-brain-study). This study comprises a pool of several thousand adults with an age of 18–60 years that declared consent to be invited to participate in health-related surveys. Subjects were postally informed about the study and asked for participation. From all contacted individuals, we received written consent from 883 valid participants that were included in the study. After receiving consent, the questionnaires were sent out to the participants, asking them to rate all questions in a single session at home and afterwards send them to our department. Exclusion criteria were active psychiatric or neurological disorders, which we assessed with a standard questionnaire from our department, asking for patient history and current symptoms of mental disorders.

### Questionnaires

All subjects cross-sectionally filled out a list of psychometric questionnaires in order to assess state boredom (*MSBS*: Multidimensional State Boredom Scale^26^), trait boredom (*BPS*: Boredom Proneness Scale^31^) and psychometric characteristics of mental health that previously showed a link to boredom (*GHQ-28*: General Health Questionnaire^62^, *CAARS:S-L*: Conner’s Adult ADHD Rating Scale^63^, *BDI-II*: Beck’s Depression Inventory^64^, I-8: Impulsivity Questionnaire^65,66^, *STAI-Y*: State Trait Anxiety Inventory^67^) as well as general information of sociodemographic background. Subjects received an expense allowance of 5€ for participation. The demographic characteristics of the study sample are depicted in Supplementary Table 1. The dataset of this study was also used as a control with healthy participants in a clinical study on boredom prevalence^68^.

#### Multidimensional state boredom scale (MSBS)

The MSBS is a 29 item questionnaire to measure state boredom^26^ on a 7-point Likert scale. It comprises subscales quantifying Disengagement, High Arousal, Low Arousal, Inattention and Time Perception. For creating a German version of the questionnaire, the authors of the initial version were contacted and asked for their agreement. After receiving consent, all items were translated from the original English questionnaire by a professional translation agency. These German translations were then back-translated to English and verified by three independent persons to show full congruence in meaning with the original questions.

#### Boredom proneness scale (BPS)

The BPS is a self-report scale to quantify individual trait boredom^31^. It includes 28 items that are rated on a 7-point Likert scale. The items 1, 7, 8, 11, 13, 15, 18, 22, 23, 24 are rated inversely, with low scores reflecting high boredom proneness. The dimensionality of the BPS is unclear, with controversial evidence for different factor structures^32,39,58^. In line with the MSBS, the translation of the BPS questions was conducted in two steps: initial translation to German and subsequent translation back to English, hence ensuring unaltered meaning of all items.

#### Sociodemographic information

Participants were asked about their date of birth, home country, current residency and psychiatric disorders in their families.

#### General health questionnaire (GHQ-28)

The GHQ-28 is a questionnaire to screen for psychiatric disorders in a population^62^. The higher an individual scores, the more likely is the presence of mental complaints. This 28-item version includes four subscales: Somatic Symptoms, Anxiety and Insomnia, Social Dysfunction and Depression.

#### Conner’s adult ADHD rating scale (CAARS-S:L)

The CAARS-S:L is the long self-report version of a questionnaire to assess symptoms of Adult Attentional Deficit Hyperactivity Disorder^63,69^. It comprises 66 items and covers subscales for Inattention/Memory Problems, Hyperactivity/Restlessness, Impulsivity/Emotional Lability and Problems with Self-Concept.

#### Beck’s depression inventory (BDI-II)

The BDI-II is a self-report inventory including 21 items, used to measure the prevalence and severity of depressive symptoms^64^. It has been established for adult persons with a minimal age of 13 years, matching this study’s sample.

#### Impulsivity questionnaire (I-8)

The I-8 is a short 8 item self-report inventory that was originally developed based on the UPPS Impulsive Behavior Scale^70,71^, in order to assess the tendency for impulsive behavior in the subdimensions Urgency, Willingness to Take Risks, Endurance and Purpose^65,66^. Note that the subscales Endurance and Purpose inversely reflect the impulsivity of individuals.

#### State trait anxiety inventory (STAI-Y)

The STAI-Y is a diagnostic tool for measuring individual symptoms of state and trait anxiety consisting of 20 items^67^. Here, we only used a subpart of the questionnaire to quantify trait anxiety.

### Statistical analysis

All analyses were conducted using the following software: IBM SPSS® Statistics for Windows (Armonk, NY: IBM Corp., version 27.0), MATLAB® statistics and machine learning toolbox (The Mathworks Inc., Natick, Massachusetts, USA, version R2022a), the freeware statistics program R (https://www.r-project.org) and RStudio (2009–2022 RStudio, PBC, version 2022.07.2 Build 576).

#### Questionnaire scores and item statistics

The self-report data was analyzed by computing the sum score for each questionnaire. Subjects that accidentally skipped single items of a questionnaire or subscale were excluded from the respective analysis. This exclusion explains deviations from the number of totally recruited participants and the reported N for the respective analyses in the results section.

For the two boredom questionnaires, MSBS and BPS, we further computed the standard deviation and difficulty of single-item ratings, in order to assess how reliably and differentially participants rated them. Difficulty was computed as

$${\text{difficulty}}\hspace{0.17em}=\hspace{0.17em}\frac{\left(sum -(N*Min\right))}{(N * (Max-Min))}$$

where *N* denotes the number of participants that rated the respective item, *sum* denotes the summed rating over all subjects and *min/max* denotes the minimal and maximal rating across subjects^35^. This difficulty score can take values from 0 to 1 in arbitrary units.

#### Exploratory factor analysis (EFA)

In order to determine the factor structure of the translated boredom questionnaires, we conducted exploratory factor analyses (EFA) with a random subsample of participants (MSBS: N = 291; BPS: N = 284)^72^. For this purpose, we applied a Kaiser–Meyer–Olkin test in order to confirm the suitability of the questionnaire data for EFA (KMO-coefficient = 0.86)^73^. Next, the number of factors for EFA was calculated with a Velicer’s minimum average partial test (MAP test)^36^. Based on the number of expected factors from the MAP test, we then conducted a principal component analysis with an oblimin rotation^74^. We then compared the loading patterns of single items and furthermore tested the questionnaire structure obtained by the EFA for its goodness with a subsequent confirmatory factor analysis and analysis of explained variance (R^2^).

#### Confirmatory factor analysis (CFA) and model comparison

With the remaining dataset that was not used for EFA (MSBS: N = 576, BPS: N = 571), we conducted a confirmatory factor analysis (CFA) in order to test the goodness of different factor models for each questionnaire. The items were assigned to the respective factors based on the results of our preceding exploratory factor analysis, assigning each item to the factor with the maximal absolute loading. We applied a Yuan-Bentler correction^75,76^ and the robust maximum likelihood estimator, comparing the different factor models in respect to their goodness-of-fit index (GFI)^77^, comparative fit index (CFI), root mean square error of approximation (RMSEA), standardized root mean square residual and Akaike information criterion (AIC)^37^.

#### Factor analysis of shortened questionnaire versions

With the subset of data that was used for our initial factor analysis (MSBS: N = 291; BPS: N = 284), we wanted to test the effect of excluding ambiguous items on the questionnaire structure. Therefore, we removed all items from our data that showed absolute loadings larger than 0.3 on more than one factor, as well as all items that only showed loadings smaller than 0.3 on all factors. For the BPS, we additionally removed all items with reverse wording^40^. For the MSBS this resulted in the items 1, 3, 4, 6, 11, 12, 14, 16, 17, 18, 19, 20, 21, 22, 24, 25, 26, 27, 28 and 29 considered in the shortened version. For the BPS this resulted in the items 3, 4, 5, 9,10, 12, 14, 16, 17, 19, 20, 21, 25, 26 and 27 considered in the shortened version. Then, we again tested the factor structure and explained variance by conducting a Velicer’s minimum average partial test^36^ and an EFA.

#### Internal consistency (self-reliability of questionnaires)

In order to probe the internal consistency of the translated boredom questionnaires, we computed Cronbach’s α^38^ for the sum score and subscale scores of each questionnaire. This score can take values between 0 and 1, where high values indicate a good internal reliability.

## Supplementary Information

Supplementary Information.

## Data Availability

The data of this study is available from the corresponding authors upon reasonable request.

## Code availability

The code to reproduce the analyses of this study is available from the corresponding authors upon reasonable request.
