# Supplementary material for: Validation of a German version of the Boredom Proneness Scale and the Multidimensional State Boredom Scale
Source: Sci Rep. 2024 Feb 5;14:2905. doi: 10.1038/s41598-024-53236-4 (PMC10844236; doi:10.1038/s41598-024-53236-4)
Supplement: Supplementary file 1 — Supplementary Information. [file 41598_2024_53236_MOESM1_ESM.pdf]

Validation of a German version of the Boredom Proneness Scale and the Multidimensional State Boredom Scale

Katharina Zerr[1,*], Johannes P.-H. Seiler[2,*], Simon Rumpel[2,**], Oliver Tüscher[1,3,4,**]

[1] Department of Psychiatry and Psychotherapy, University Medical Center of the Johannes Gutenberg University Mainz, Untere Zahlbacher Straße 8, 55131 Mainz, Germany

[2] Institute for Physiology, University Medical Center of the Johannes Gutenberg University Mainz, Hanns-Dieter-Hüsch-Weg 19, 55131 Mainz, Germany

[3] Leibniz Institute for Resilience Research, Wallstraße 7, 55122 Mainz, Germany

[4] Institute of Molecular Biology, Ackermannweg 4, 55128 Mainz, Germany

[*] First authors that contributed equally

[**] Last authors that contributed equally

Correspondence concerning this article should be addressed to Johannes Seiler, Institute of Physiology, Focus Program Translational Neurosciences, University Medical Center of the Johannes Gutenberg University Mainz, Hanns-Dieter-Hüsch-Weg 19, 55131 Mainz, Germany. E-mail: johseile@uni-mainz.de .

Additional information

German version of the Multidimensional State Boredom Scale

1. Die Zeit scheint langsamer zu vergehen als sonst.
2. Ich stecke in einer Situation fest, die bedeutungslos ist.
3. Ich bin leicht abgelenkt.
4. Ich fühle mich einsam.
5. Alles um mich herum ist mir gerade lästig.
6. Ich wünschte, die Zeit würde schneller vergehen.
7. Alles erscheint mir wiederholend und gleichförmig.
8. Ich fühle mich bedrückt.
9. Ich fühle mich zu Taten gedrängt, die keinerlei Bedeutung für mich haben.
10. Ich fühle mich gelangweilt.
11. Die Zeit zieht sich.
12. Ich bin launischer als sonst.
13. Ich bin unentschieden und unsicher, was ich als nächstes tun soll.
14. Ich fühle mich unruhig.
15. Ich fühle mich leer.
16. Es fällt mir schwer, meine Aufmerksamkeit zu fokussieren.
17. Ich möchte etwas Unterhaltsames tun, doch weiß ich nicht, was mich reizen würde.
18. Die Zeit vergeht sehr langsam.
19. Ich wünschte, ich würde etwas Aufregenderes tun.
20. Meine Aufmerksamkeitsspanne ist kürzer als sonst.
21. Ich bin gerade ungeduldig.
22. Ich verschwende gerade Zeit, die ich sonst besser nutzen könnte.

23. Meine Gedanken schweifen umher.

24. Ich möchte, dass etwas passiert, doch kann ich nicht genau sagen was.

25. Ich fühle mich vom Rest der Welt abgeschnitten.

26. Gerade scheint die Zeit langsam zu vergehen.

27. Die Menschen um mich herum verärgern mich.

28. Ich fühle mich, als würde ich nur herumsitzen und darauf warten, dass etwas passiert.

29. In meinem Umfeld gibt es niemanden, um mich zu unterhalten.

German version of the Boredom Proneness Scale

1. Es fällt mir leicht, mich auf meine Aktivitäten zu konzentrieren.
2. Beim Arbeiten an einer Sache bin ich oft mit anderen Dingen beschäftigt.
3. Die Zeit scheint langsam zu vergehen.
4. Ich weiß oft nicht, womit ich mich beschäftigen kann.
5. Ich bin oft in Situationen gefangen, in denen ich bedeutungslose Dinge tue.
6. Die Urlaubsfotos von jemand anderem anzusehen langweilt mich enorm.
7. Mir kommen immer Ideen in den Sinn, was ich tun kann.
8. Ich finde es leicht, mich selbst zu unterhalten.
9. Viele Dinge, die ich tun muss wiederholen sich und sind monoton.
10. Es braucht mehr Anreize, um mich zu Tätigkeiten zu bewegen als bei den meisten anderen Leuten.
11. Die meisten Dinge, die ich tue verleihen mir einen Kick.
12. Ich bin selten von meiner Arbeit begeistert.
13. In jeder Situation finde ich in der Regel etwas, das mich interessiert.
14. Einen großen Teil meiner Zeit sitze ich nur herum und tue nichts.
15. Ich bin gut im geduldigen Warten.
16. Ich habe oft zu viel Zeit zur Verfügung ohne etwas zu tun zu haben.
17. Situationen, in denen ich warten muss machen mich rastlos.
18. Ich wache oft mit einer neuen Idee auf.
19. Es wäre schwer für mich, eine Arbeit zu finden, die mich genug begeistert.
20. Ich hätte gerne mehr fordernde Aufgaben in meinem Leben.
21. Ich habe das Gefühl, die meiste Zeit unter meinen Möglichkeiten zu arbeiten.
22. Viele Leute würden sagen, ich bin eine kreative, fantasievolle Person.
23. Ich habe so viele Interessen, dass ich gar nicht genug Zeit für alle habe.
24. Unter meinen Freunden bleibe ich am längsten bei Tätigkeiten.

25. Ohne etwas Aufregendes, sogar Gefährliches zu tun fühle ich mich halb tot und matt.

26. Es braucht eine ganze Menge an Abwechslung, um mich glücklich zu halten.

27. Es scheint mir, dass im Fernsehen und Kino immer das Gleiche läuft; es ödet mich an.

28. Als ich jung war, fand ich mich oft in monotonen, ermüdenden Situationen wieder.

Supplementary Table 1

	Healthy cohort (n = 883)
**Gender**	
Male	297 (33.6%)
Female	586 (66.4%)
**Age** (years)	
Mean	41.5
Standard deviation	11.72
**Home continent/country**	
Europe/Germany	808 (93.4%)
Europe/other	34 (4.0%)
Asia	17 (2.0%)
North America	2 (0.2%)
Middle America	1 (0.1%)
South America	2 (0.2%)
Africa	1 (0.1%)
**Psychiatric disorder in family**	
Yes	285 (32.3%)

Supplementary Table 1 – Demographic characteristics of the study sample

Supplementary Table 2

	Number of items	Number of subjects	Cronbach's α
**GHQ-28**	28	873	0.926
**BDI-II**	21	878	0.905
**CAARS:S-L**	66	827	0.945
**STAI-Y**	20	871	0.939
**I-8 Urgency**	2	877	0.728
**I-8 Purpose**	2	878	0.853
**I-8 Endurance**	2	877	0.682
**I-8 Risk**	2	878	0.932

Supplementary Table 2 – Internal consistency of self-report assessments in the study

Supplementary Table 3

MSBS item	N	Min.	Max.	Sum	Mean	SD	Difficulty
**1**	882	1	7	1947	2.21	1.445	0.201
**2**	883	1	7	1631	1.85	1.317	0.141
**3**	881	1	7	2839	3.22	1.604	0.370
**4**	883	1	7	2024	2.29	1.579	0.215
**5**	882	1	7	2177	2.47	1.495	0.245
**6**	883	1	7	1568	1.78	1.362	0.129
**7**	882	1	7	2108	2.39	1.519	0.232
**8**	883	1	7	2177	2.47	1.596	0.244
**9**	883	1	7	1776	2.01	1.387	0.169
**10**	883	1	7	1964	2.22	1.484	0.204
**11**	883	1	7	1666	1.89	1.284	0.148
**12**	882	1	7	2282	2.59	1.688	0.265
**13**	883	1	7	2357	2.67	1.664	0.278
**14**	883	1	7	2544	2.88	1.737	0.314
**15**	882	1	7	1804	2.05	1.517	0.174
**16**	882	1	7	2418	2.74	1.605	0.290
**17**	883	1	7	2209	2.50	1.655	0.250
**18**	883	1	7	1586	1.80	1.200	0.133
**19**	883	1	7	2592	2.94	1.751	0.323
**20**	882	1	7	2321	2.63	1.663	0.272
**21**	883	1	7	2427	2.75	1.686	0.291
**22**	883	1	7	2477	2.81	1.786	0.301
**23**	882	1	7	2816	3.19	1.761	0.365
**24**	883	1	7	2246	2.54	1.724	0.257
**25**	882	1	7	1829	2.07	1.489	0.179
**26**	881	1	7	1698	1.93	1.340	0.155
**27**	882	1	7	2112	2.39	1.519	0.232
**28**	879	1	7	1882	2.14	1.557	0.190
**29**	881	1	7	1402	1.59	1.142	0.099

Supplementary Table 3– Item statistics for the Multidimensional State Boredom Scale: Most items show intermediate difficulty and standard deviation, indicating that they were rated differentially but reliably.

Supplementary Table 4

MSBS item	Factor		
	1	2	3
**1**	0.08	-0.17	**0.79**
**2**	0.00	**0.55**	0.27
**3**	**0.63**	-0.06	0.17
**4**	0.12	**0.65**	-0.06
**5**	**0.37**	**0.45**	-0.08
**6**	-0.05	0.18	**0.50**
**7**	-0.05	**0.59**	0.11
**8**	**0.46**	0.40	-0.02
**9**	0.18	**0.52**	-0.03
**10**	-0.14	**0.62**	0.27
**11**	-0.02	0.15	**0.78**
**12**	**0.53**	0.22	0.03
**13**	**0.47**	0.27	0.03
**14**	**0.73**	0.11	-0.03
**15**	0.34	**0.55**	-0.06
**16**	**0.80**	-0.09	0.10
**17**	0.19	**0.55**	0.06
**18**	-0.02	0.07	**0.87**
**19**	0.05	**0.50**	0.14
**20**	**0.67**	0.06	0.08
**21**	**0.67**	0.05	-0.06
**22**	**0.46**	0.18	0.10
**23**	**0.76**	-0.05	0.04
**24**	0.23	**0.50**	0.14
**25**	0.22	**0.52**	-0.03
**26**	0.11	-0.02	**0.79**
**27**	**0.33**	0.28	0.00
**28**	0.08	**0.52**	0.27
**29**	-0.16	**0.65**	0.07

Supplementary Table 4– Exploratory factor analysis of the German MSBS, loadings on a suggested 3-factor structure: We applied an exploratory factor analysis and tested the loadings of each item on the three factors, suggested by a Velicer's minimum average partial test (see Methods, data from N=291 participants). Loadings >0.3 are presented in bold letters.

Supplementary Table 5

MSBS N=867		Diseng.	High Arousal	Low Arousal	Inattent.	Time Percept.
Sum Score	SpearmanR	**0.936**	**0.862**	**0.833**	**0.849**	**0.662**
	Sig. (p)	<0.001	<0.001	<0.001	<0.001	<0.001
Diseng.	SpearmanR	-	**0.726**	**0.730**	**0.755**	**0.602**
	Sig. (p)		<0.001	<0.001	<0.001	<0.001
High Arousal	SpearmanR		-	**0.709**	**0.748**	**0.429**
	Sig. (p)			<0.001	<0.001	<0.001
Low Arousal	SpearmanR			-	**0.627**	**0.423**
	Sig. (p)				<0.001	<0.001
Inattent.	SpearmanR				-	**0.486**
	Sig. (p)					<0.001

Supplementary Table 5– Inter-correlations of the MSBS sum score and the MSBS subscales: Significant Spearman correlations after Bonferroni correction for multiple testing are highlighted in bold letters. Redundant or self-correlations are left empty.

Supplementary Table 6

BPS item	N	Min.	Max.	Sum	Mean	SD	Difficulty
**1**	881	1	7	2677	3.04	1.526	0.340
**2**	882	1	7	3063	3.47	1.607	0.412
**3**	882	1	7	1696	1.92	1.279	0.154
**4**	881	1	7	1696	1.93	1.313	0.154
**5**	882	1	7	2028	2.30	1.530	0.217
**6**	882	1	7	2724	3.09	1.759	0.348
**7**	882	1	7	2378	2.70	1.492	0.283
**8**	881	1	7	2171	2.46	1.399	0.244
**9**	882	1	7	2869	3.25	1.575	0.375
**10**	881	1	7	1929	2.19	1.425	0.198
**11**	881	1	7	4104	4.66	1.457	0.610
**12**	882	1	7	2447	2.77	1.559	0.296
**13**	881	1	7	2551	2.90	1.411	0.316
**14**	881	1	7	1597	1.81	1.299	0.135
**15**	882	1	7	3616	4.10	1.847	0.517
**16**	882	1	7	1834	2.08	1.479	0.180
**17**	882	1	7	2805	3.18	1.730	0.363
**18**	882	1	7	3691	4.18	1.631	0.531
**19**	880	1	7	2257	2.56	1.619	0.261
**20**	882	1	7	2679	3.04	1.726	0.340
**21**	880	1	7	2876	3.27	1.828	0.378
**22**	880	1	7	3022	3.43	1.718	0.406
**23**	882	1	7	2966	3.36	1.869	0.394
**24**	871	1	7	3459	3.97	1.426	0.495
**25**	881	1	7	1572	1.78	1.227	0.131
**26**	881	1	7	2422	2.75	1.637	0.292
**27**	880	1	7	3236	3.68	1.926	0.446
**28**	880	1	7	1680	1.91	1.329	0.152

Supplementary Table 6– Item statistics for the Boredom Proneness Scale: Most items show intermediate difficulty and standard deviation, indicating that they were rated differentially but reliably.

Supplementary Table 7

BPS item	Factor		
	**1**	**2**	**3**
**1**	0.05	0.24	**0.41**
**2**	0.19	-0.01	0.27
**3**	**0.39**	0.05	0.18
**4**	**0.47**	0.16	0.15
**5**	**0.70**	0.01	0.17
**6**	0.25	-0.07	0.15
**7**	-0.03	**0.74**	-0.03
**8**	0.03	**0.63**	0.25
**9**	**0.55**	-0.04	-0.04
**10**	**0.44**	0.16	0.19
**11**	0.17	**0.54**	-0.23
**12**	**0.61**	0.13	-0.07
**13**	0.12	**0.68**	0.15
**14**	**0.49**	0.18	0.19
**15**	-0.10	0.17	**0.38**
**16**	**0.46**	0.20	0.00
**17**	0.03	0.13	**0.53**
**18**	0.03	**0.60**	-0.13
**19**	**0.54**	0.14	0.09
**20**	**0.69**	0.00	-0.07
**21**	**0.77**	-0.16	-0.11
**22**	-0.10	**0.57**	-0.06
**23**	0.04	**0.63**	-0.12
**24**	-0.05	**0.41**	0.00
**25**	0.08	-0.22	**0.54**
**26**	0.08	-0.10	**0.62**
**27**	**0.35**	-0.08	0.05
**28**	0.23	0.07	0.24

Supplementary Table 7– Exploratory factor analysis of the German BPS, loadings on a suggested 3-factor structure: We applied an exploratory factor analysis and tested the loadings of each item on the three factors, suggested by a Velicer's minimum average partial test (see Methods, data from N=284 participants). Loadings >0.3 are presented in bold letters.
